# The Analysis of Correlative Factors of Visual Acuity with Intravitreal Conbercept Injection in Macular Edema Associated with Branch Retinal Vein Occlusion

**DOI:** 10.1155/2018/7348153

**Published:** 2018-11-12

**Authors:** Wenjuan Luo, Fengjiao Jia, Min Liu, Yunxiao Wang, Ting Zhang

**Affiliations:** ^1^Affiliated Hospital of Qingdao University, Medical College, Qingdao, China; ^2^The People's Hospital of Zoucheng City, Jining, China; ^3^Municipal Hospital of Qingdao City, Qingdao, China

## Abstract

**Purpose:**

To evaluate the correlative factors of best-corrected visual acuity (BCVA) with intravitreal conbercept injection in macular edema secondary to branch retinal vein occlusion (BRVO).

**Methods:**

This is a self-controlled retrospective study. 35 eyes of 35 patients with macular edema secondary to BRVO were enrolled. After an initial intravitreal injection of conbercept (0.5 mg/0.05 ml) monthly up to 3 months, a pro re nata (PRN) strategy was adopted based on the increase in central foveal thickness (CFT). Data collected at various time points include BCVA, CFT, photoreceptor layer thickness (PLT), and outer nuclear layer thickness (ONLT) on optical coherence tomography. The correlation between CFT, PLT, ONLT, and BCVA before and after injections was analyzed.

**Results:**

Compared with baseline, in months 1, 3, and 6 after injection, the improvement of BCVA was 20.63, 22.94, and 21.06 ETDRS letters, respectively (*F*=195.843, *P* < 0.01), and the decrease of CFT was 217.37 *μ*m, 224.57 *μ*m, and 224.06 *μ*m, respectively (*F*=148.522, *P* < 0.01). The PLT in months 1, 3, and 6 after therapy has significant improvement of 11.14 *μ*m, 13.03 *μ*m, and 13.49 *μ*m (*F*=64.116, *P* < 0.01), while the ONLT has a significant decrease of 225.29 *μ*m, 237.66 *μ*m, and 239.11 *μ*m, respectively (*F*=145.231, *P* < 0.01). The changes in the treatment group were significant in different periods. The mean number of injections was 3.26 ± 0.50 from baseline to month 6.

**Conclusions:**

Intravitreal injection of conbercept provides an effective treatment for macular edema due to BRVO. With six-month treatment, there were a positive correlation between BCVA and PLT (*r*=0.592, *P* < 0.001), a negative correlation between BCVA and ONLT (*r*=−0.480, *P*=0.005), and no correlation between BCVA and CFT (*P*=0.506).

## 1. Introduction

Retinal vein occlusion (RVO) is the second most common retinal vascular disorder after diabetic retinopathy. RVO is an important cause of visual impairment [1]. The main complications of branch retinal vein occlusion (BRVO) include macular edema (ME) and retinal neovascularization. The persistent macular edema (ME) is the primary cause of visual impairment [2]. In recent years, the researchers on the ME associated with BRVO have a further understanding and views. On the one hand, thrombosis of the retinal veins causes an increase in retinal capillary pressure, resulting in increased capillary permeability and leakage of fluid and blood into the retina. On the other hand, retinal nonperfusion and ischemia increase the levels of vascular endothelial growth factors (VEGFs), and the high level of VEGF may in turn promote the retinal nonperfusion and ischemia [2]. Hence, exacerbating ME and retinal neovascularization leads to visual impairment. In clinical work, the treatment modality is reducing macular edema, partial retinal ischemia, and cellular injury and improving visual acuity in BRVO. At present, the major treatment options of macular edema caused by BRVO include intravitreal injection of triamcinolone acetonide and antivascular endothelial growth factor agents, dexamethasone intravitreal implants, focal/grid or panretinal photocoagulation, and surgical interventions.

Optical coherence tomography (OCT) is often used to detect changes within the retinal architecture and to quantify retinal thickness and can be used to diagnose retinal diseases. Compared with conventional time-domain optical coherence tomography (OCT), spectral-domain optical coherence tomography (SD-OCT) yields a high axial resolution and provides cross-sectional images of tissue structures on the micron scale, including the photoreceptor inner and outer segment junction (IS/OS) and the external limiting membrane (ELM) [3–5].

Histologic studies of eyes with retinal vein occlusion showed that cystoid spaces in ME associated with BRVO often in the outer nuclear layer and causes liquefaction necrosis of cells, which results in photoreceptor cell loss and dysfunction in the fovea [6]. In a study by Ota et al. [6], integrity of the photoreceptor layer in the fovea is associated with BCVA in resolved ME, and the change in PLT is more significant than the decrease in the CFT on the improvement of visual function.

Recently, antivascular endothelial growth factor (VEGF) therapy, including intravitreal ranibizumab (Lucentis), aflibercept (Eylea), and bevacizumab (Avastin), has become the option of first choice at home and abroad because the high level of VEGF may promote the retinal nonperfusion and ischemia [7]. Conbercept (Lumitin), one of the anti-VEGF medications, is a fusion protein composed of extracellular domain 2 of VEGF receptor 1 and extracellular domains 3 and 4 of VEGF receptor 2 combined with the Fc portion of human immunoglobulin G1. It shows a very high affinity for placental growth factor and all types of VEGF and can rapidly block VEGF-A in a long time, which makes the blood-retina barrier recover, central retinal thickness (CFT) decrease, and visual function of patients with BRVO significantly improve. Preclinical experimental studies have demonstrated the greater binding affinity of conbercept for VEGF than bevacizumab, ranibizumab, or aflibercept. And conbercept has been approved by the China Food and Drug Administration for the treatment of wet age-related macular degeneration (wAMD) and can be used in clinic. The present study has confirmed that intravitreal conbercept injection can resolve ME secondary to BRVO and improve visual function [2]. But research on the efficacy and safety of therapy on ME secondary to BRVO is limited. In this study, we assess the efficacy and safety of intravitreal conbercept injection on ME secondary to BRVO, including best-corrected visual acuity (BCVA), the CFT, fundus photography, and fundus fluorescein angiography (FFA). We also evaluated the correlation of best-corrected visual acuity (BCVA) and changes in various retinal layers' thickness in resolved ME.

## 2. Materials and Methods

In this retrospective study, we reviewed the medical records of 35 patients (16 males, 14 females), who had central macular edema (CME) secondary to BRVO and who were first seen at the Affiliated Hospital of Qingdao University between December 2014 and June 2016. All patients received intravitreal conbercept injection for at least 6 months, and the first treatment to the CME onset is 7 d to 12 mo.

### 2.1. Participants

The study included patients ≥18 years with central macular edema secondary to BRVO diagnosed by OCT and FFA within 12 months, and the study eye's BCVA was between 19 and 73 Early Treatment Diabetic Retinopathy Study (ETDRS) letters (20/400 and 20/40 Snellen equivalent), and the other eye's BCVA was >34 ETDRS letters (>20/200 Snellen equivalent). Only one eye from each patient was included in this study.

The exclusion criteria included ocular or general use of other test drugs, systemic steroids within the last 6 months for at least 30 days, intraocular or periocular steroid treatment in the study eye within the last 3 months, eyes with a history of treatment that could potentially affect the study, including intraocular laser and vitrectomy, and combined other ocular or systemic problems including ocular inflammation in both eyes, uncontrolled glaucoma, myocardial infarction, and uncontrolled hypertension.

### 2.2. Treatments

**Figure 1:**
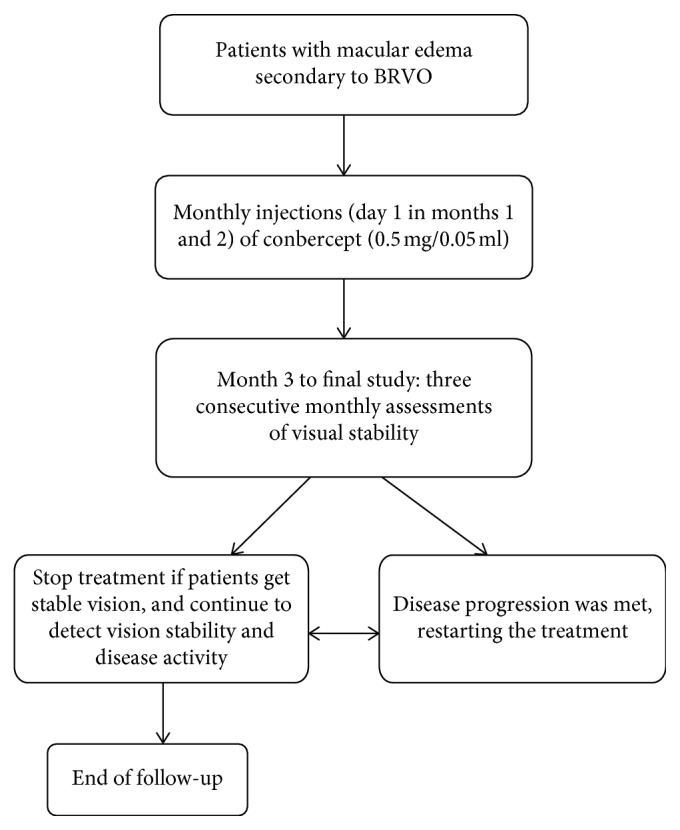
Study flow chart.

In all 35 patients, intraocular injections of conbercept (0.5 mg/0.05 ml) were administered monthly in the loading phase of 3 months. From month 3, the patients received injection as needed or pro re nata (PRN), and all patients were evaluated monthly. If CFT ≥ 250 *μ*m on optical coherence tomography and visual acuity decreased because of disease progression, treatment was restarted for eyes. The final study visit until stable visual acuity is shown in Figure 1.

All the injections were administered in a sterile operating room under a topical anesthesia, i.e., oxybuprocaine hydrochloride ophthalmic solution. Intravitreal conbercept injection 0.5 mg/0.05 ml was given by one retinal specialist. The patients were treated with a topical ophthalmic antibiotic four times a day for 3 days before and after the injection. All patients were monitored for any local or systemic adverse effects throughout the study.

### 2.3. Outcome Measurements

The primary efficacy outcome measure was the mean changes from baseline in BCVA at 1, 3, and 6 months. The secondary outcome was the mean changes from baseline in CFT, PLT, and ONLT at 1, 3, and 6 months. Safety assessments included ocular and nonocular adverse events (AEs) and serious AEs (SAEs).

The BCVA, CFT, PLT, and ONLT were evaluated at baseline and months 1, 3, and 6, respectively. BCVA was assessed following the ETDRS protocol [8]. The CFT, PLT, and ONLT were evaluated with spectral-domain optical coherence tomography. Fundus photography (AFC-330; Nidek, Japan) and FFA (HRA SPECTRLIS; Heidelberg, German) were performed at baseline and months 1, 3, and 6, respectively. The patients had retinal laser when FFA showed there were nonperfusion areas ≥ 4 PD.

### 2.4. Statistical Analyses

The data of this study were analyzed using SPSS v.22.0 for Windows software (SPSS, Chicago, IL), and all statistical tests were two-sided. A *P* value of <0.05 was considered statistically significant. Efficacy outcome measures were analyzed in the full analysis set, which comprised all eyes that received at least three injections and had a baseline and ≥1 postbaseline BCVA assessment. Independent-samples *T* test was used for baseline data comparison. The changes in BCVA, CFT, ONLT, and PLT between the follow-up and the baseline were evaluated by one-way repeated-measures ANOVA at a two-sided significance level of 5%. All of measurement data were examined using the Shapiro–Wilk test. The correlation assessments of BCVA, CFT, PLT, and ONLT were using Pearson's analysis. The safety analysis set included all patients who received study treatment.

## 3. Results

### 3.1. Patient Demographics and Baseline Characteristics

The 35 patients in this study included 16 (47.22%) men and 19 (52.78%) women, with a mean age of 54.17 ± 11.18 years (range 30–71 years) at the time of their first conbercept injection. In those patients, 15 (41.66%) of them were with hypertension, 6 (16.68%) of them were with diabetes, and 15 (41.66%) of them were without any disease (Table 1).

**Figure 2:**
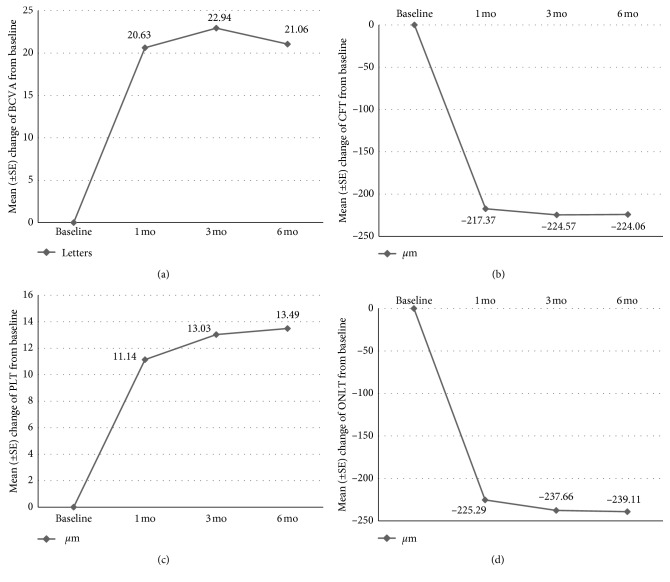
The mean change in BCVA (a), CFT (b), PLT (c), and ONLT (d) from baseline to month 6.

The mean BCVA was 50.89 ± 9.21 letters at baseline and improved to 71.51 ± 10.79 letters at 1 month after injection, with a mean increase of 20.63 letters (*p* < 0.001). The mean BCVA was 73.83 ± 10.33 letters at 3 months after injection, with a mean increase of 22.94 letters (*p* < 0.001). The mean BCVA was 71.94 ± 10.04 letters at 6 months after injection, with a mean increase of 21.06 letters (*p* < 0.001) (Figure 2). One-way repeated measures ANOVA showed that there was a significant difference in BCVA between baseline and every other month. In this study, the mean central foveal thickness was 473.71 ± 78.33 *μ*m at baseline and significantly reduced to 256.34 ± 66.60 *μ*m, with a mean reduction of 217.37 *μ*m (*p* < 0.001) at 1 month, 249.14 ± 54.41 *μ*m, a mean reduction of 224.57 *μ*m (*p* < 0.001) at 3 months, 249.66 ± 33.55 *μ*m, and a mean reduction of 224.06 *μ*m (*p* < 0.001) at 6 months (Figure 2). There was a significant difference between baseline and every other month. FFA outcomes were evaluated and included variable venous filling, blockage by blood, hyperfluorescence due to leakage, and retinal neovascularization.

All patients who received ≥3 injections of conbercept were evaluated for safety. From baseline to month 6, two patients had subconjunctival hemorrhage and completely absorbed in 2 weeks. 3 patients experienced temporary high IOP, the mean IOP was 32.3 mmHg, and they were managed successfully without any treatment. There were no cases of retinal detachment, endophthalmitis, glaucoma, cataract progression, iris neovascularization, and any nonocular AEs in all 35 patients.

### 3.2. Correlation

**Figure 3:**
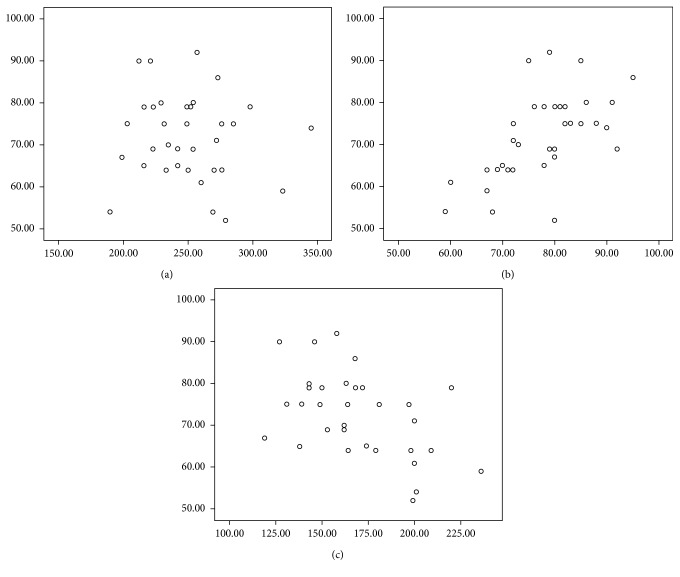
The scatter plots for CFT, PLT, ONLT, and BCVA: (a) the *x*-axis represents CFT and *y*-axis represents BCVA; (b) the *x*-axis represents PLT and *y*-axis represents BCVA; (c) the *x*-axis represents ONLT and the *y*-axis represents BCVA.

The mean PLT was 64.46 ± 12.20 *μ*m at baseline, and the respective values for the mean improvement in PLT in months 1, 3, and 6 were 11.14 *μ*m, 13.03 *μ*m, and 13.49 *μ*m (Figure 2). There was statistical significance (*p* < 0.001) between the baseline of PLT and months 1, 3, and 6. A similar trend was seen in the ONLT. The mean ONLT was 409.43 ± 78.52 *μ*m at baseline, and the respective values for the mean decrease in ONLT in months 1, 3, and 6 were 225.29 *μ*m, 237.66 *μ*m, and 239.11 *μ*m (Figure 2). There was statistical significance (*p* ≤ 0.001) between the baseline of ONLT and months 1, 3, and 6. With treatment of intravitreal conbercept injection, BCVA correlated with PLT and ONLT (*r*=0.592, *p* < 0.001, and *r*=−0.480, *p* ≤ 0.005, respectively), whereas BCVA was not correlated with CFT after treatment (*p* > 0.5) (Table 2; Figure 3).

## 5. Discussion

The intravitreal injection of conbercept (IVC) has proven to be effective in reducing macular edema and improving visual acuity in patients with BRVO [2]. Many studies have shown that macular edema could cause serious cone dysfunction [8, 9]. The normal function of the foveal photoreceptor layer may well be necessary to regain good visual function. Therefore, some researchers tried to measure the thickness of various retinal layers, in order to lay a theoretical foundation for the visual recovery of patients with macular edema [4, 5, 10]. In this study, we evaluated the change in various retinal layers' thickness in BRVO after conbercept injection and analyzed the correlation between them and BCVA.

In the current study, the primary efficacy end point of the change was BCVA at month 3 with conbercept injections, and all secondary efficacy end points include change in BCVA and the thickness of various retinal layers. Treatment with monthly IVC over 3 months resulted in quick and sustained improvements in visual acuity and anatomic end points. These improvements were maintained and even increased, after PRN dosing with monthly evaluations through month 6. For patients with BRVO, the BCVA got the best effective point for the first time at month 3, and a mean increase of 22.94 letters in BCVA and a mean decrease of 224.57 *μ*m in CFT were gained compared to baseline. At month 6, a mean increase of 21.06 letters in BCVA and a mean decrease of 224.06 *μ*m in CFT were gained compared with baseline. It showed that IVC in macular edema secondary to BRVO has a significant effect, and the efficacy of conbercept has been greatly maintained to the 6th month. The treatment regimen was monthly IVC for 3months followed by IVC PRN (3 + PRN) in this study, but the trend of improvement in visual acuity with conbercept is similar to that with ranibizumab in the BRAVO study [11] and the CRUISE trial [12] as well as to that with aflibercept in the VIBRANT study [13] and the GALILEO study [14], all of which suggest that the effect of anti-VEGF agents on macular edema secondary to RVO occurs very soon after the initial treatment. The pathological changes in BRVO, like superficial hemorrhages, cotton wool spots, retinal edema, and capillary nonperfusion, have been improved.

The normal function of the foveal photoreceptor layer may well be necessary to regain good visual function. So, in the study, we analyzed the correlation between CFT, PLT, ONLT, and BCVA. We measured the thickness of CFT, PLT, and ONLT before and after treatment, and there was statistical significance between the baseline of CFT and months 1, 3, and 6. A similar trend was seen in the PLT and ONLT. However, we analyzed the correlation between the thickness of foveal retinal layers and BCVA after treatment and found that changes in PLT and ONLT were of more clinical value for improving visual function than changes in CFT. After macular edema regressed, there were a positive correlation between BCVA and PLT (*r*=0.592, *p* ≤ 0.001), a negative correlation between BCVA and ONLT (*r*=−0.480, *p* ≤ 0.005), and no correlation between BCVA and CFT (*p* > 0.5).

It is generally believed that the regression of ME is necessary for recovery of the lost visual acuity due to ME associated with BRVO. However, only some patients do achieve full recovery of vision after regression of ME. Other patients achieve limited visual improvement despite successful regression of ME. In histology, macular edema was noted mostly in the outer nuclear layer, causing liquefaction necrosis of photoreceptor cells, and led to population losses and dysfunction of photoreceptor cells in the macular central fovea. Although, retinal thickness has been reduced to physiological levels successfully after regression of macular edema, the loss of photoreceptor cells may lead to limited visual recovery. Altunel et al. [4] and Ota et al. [5] showed that there was no correlation between CFT and BCVA after treatment. The outcome of our study in correlation between BCVA and CFT with conbercept treatment is similar to theirs.

We hypothesized that the acute phase of macular edema secondary to BRVO may result in structural damages, disorganization, and loss of outer photoreceptor cells. On the contrary, severe ischemia and hypoxia may directly lead to the death of photoreceptor cells. Correspondingly, we observed the increased thickness of the outer nuclear layer because of increased oozing and the reduced thickness of the photoreceptor layer because of cell injury on OCT before treatment. Following treatment, macular edema subsided, function of photoreceptor cell recovered or reconstructed, the thickness of the outer nuclear layer decreased, and the thickness of the photoreceptor layer increased. Meanwhile, there were a positive correlation between BCVA and PLT and a negative correlation between BCVA and ONLT in our measurement and analysis. So we can predict the final efficacy of visual acuity by PLT and ONLT when we treat patients who had macular edema secondary to BRVO.

No unexpected safety findings were reported. The safety outcomes were consistent with those reported in studies of conbercept in wet AMD [2]. During the study, there were 2 cases of subconjunctival hemorrhage (completely absorbed after 2 weeks) and 3 cases of temporary high IOP (IOP returned to normal after 1 day).

Our study posed three limitations. First, our sample size was relatively small. Second, BRVO is a chronic disease, and the study is a stage study; 6-month observation is short so it is difficult to fully assess its prognosis. Third, we manually measured the various retinal layers' thickness, and automated software would have allowed a more objective evaluation and erased any potential bias.

In conclusion, the study demonstrated efficacy of IVC in the treatment of macular edema due to BRVO and was generally well tolerated. PLT is associated with the visual function with ME due to BRVO after IVC. Also, ONLT seems to be more closely related to visual acuity improvement than to CFT decrement.

## Figures and Tables

**Table 1:** Patient demographics and baseline characteristic efficacy and safety.

Number of patients	Total
Gender	
Male, *n* (%)	16 (47.22%)
Female, *n* (%)	19 (52.78%)
Mean age (years)	
Mean ± SD	53.94 ± 11.26
Mean duration (days)	
Mean ± SD	73.11 ± 65.03
Mean BCVA (ETDRS letters)	
Mean ± SD	50.89 ± 9.21
Mean CFT (*μ*m)	
Mean ± SD	473.71 ± 78.33
Mean PLT (*μ*m)	
Mean ± SD	64.46 ± 12.20
Mean ONLT (*μ*m)	
Mean ± SD	409.43 ± 78.52

**Table 2:** The correlation of BCVA with CFT, PLT, and ONLT at month 6.

	BCVA	
	*r*	*p*

CFT	−0.116	0.506
PLT	0.592	0.001
ONLT	−0.480	0.005

## Data Availability

The clinical data used to support the findings of this study are available from the corresponding author upon request.
